# Extracellular vesicles in tumorigenesis, metastasis, chemotherapy resistance and intercellular communication in osteosarcoma

**DOI:** 10.1080/21655979.2022.2161711

**Published:** 2023-06-28

**Authors:** Yi Liao, Qian Yi, Jinglong He, Dixi Huang, Jianyi Xiong, Wei Sun, Weichao Sun

**Affiliations:** a Department of Thoracic Surgery, Southwest Hospital, Army Medical University, Chongqing, Chongqing, China; b Department of Physiology, School of Basic Medical Science, Southwest Medical University, Luzhou, Sichuan, China; c The Central Laboratory, Affiliated Hospital of Putian University, Putian, Fujian, China; d Department of Orthopaedics, Shenzhen Second People’s Hospital (The First Affiliated Hospital of Shenzhen University), Shenzhen, China; e Guangzhou Medical University, Guangzhou, China

**Keywords:** Osteosarcoma, extracellular vesicles, metastasis, tumor microenvironment, therapeutics, drug resistance

## Abstract

Osteosarcoma, the most common primary malignant tumor of bone, occurs frequently in teenagers and is the third most common type of cancer among children and adolescents. Osteosarcoma originates from malignant mesenchymal stem cells. Fibroblasts, osteoblasts, osteoclasts, and immune cells, among other cell types, are present in the osteosarcoma microenvironment and work in conjunction to regulate osteosarcoma tumorigenesis. Extracellular vesicles (EVs) are spherical bilayer-membrane vesicles released by cells into extracellular spaces and body fluids, including plasma and synovial fluid. EV cargos contain various biomolecules, such as DNA, mRNAs, non-coding RNAs, proteins, lipids, and metabolites. EVs mediate intercellular communication by delivering these bioactive molecules to recipient cells and play a critical role in maintaining cellular homeostasis and promoting pathological progression. A growing body of evidence indicates that EVs play an important role in osteosarcoma tumorigenesis. In this review, we discuss existing knowledge on the important roles of tumor-derived or microenvironment-derived EVs in tumorigenesis, development, metastasis, drug resistance, and communication with the osteosarcoma microenvironment. We propose the potential of EVs in osteosarcoma diagnosis and treatment.

**Highlights:**

- Extracellular vehicles play crucial function in osteosarcoma tumorigenesis.
- Extracellular vehicles mediated the intercellular communication of osteosarcoma cells with other types cells in tumor microenvironment.
- Extracellular vehicles have potential utility in osteosarcoma diagnosis and treatment.

## GRAPHICAL ABSTRACT

**Figure:**
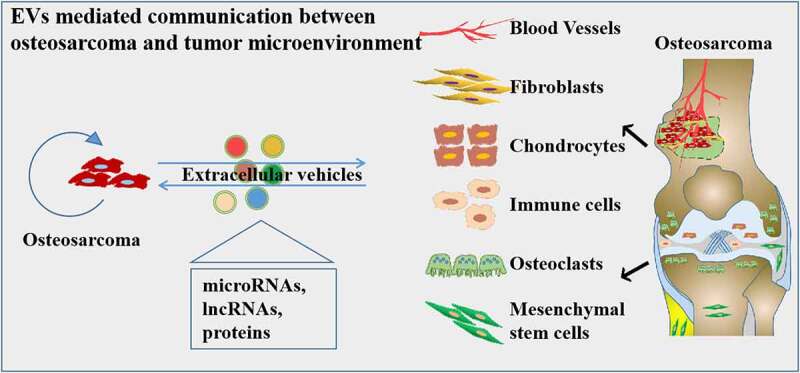

## 1. Introduction

Osteosarcoma (OS) is the most common primary malignant tumor of the bone, which occurs frequently among adolescents and is the third most common type of cancer in children and adolescents [1,2]. The incidence of osteosarcoma is 5.6 cases per million in children under the age of fifteen years [3]. The high malignancy in osteosarcoma has been attributed to the high propensity of invasiveness, disease recurrence, and chemotherapy resistance [4]. Almost 50% patients exhibit local invasion or metastasis to the lung and bone at diagnosis [5]. Although several strategies have been applied for osteosarcoma treatment in clinical settings, the prognosis of metastatic and recurrent osteosarcoma has remained stagnant in recent decades. The pathological process of osteosarcoma is extremely complex. Osteosarcoma has been shown to originate from malignant mesenchymal stem cells (MSCs) [6,7]. The initiation of tumorigenesis is based on not only the malignant transformation of cells but also the functional alteration and communication in the tumor microenvironment [8,9]. The microenvironment of osteosarcoma contains osteoblasts, osteoclasts, endothelial cells, blood vessels, tumor-associated fibroblasts, and MSCs [10]. Recent, studies have shown that extracellular vehicles (EVs), such as exosomes, play a significant role in tumor progression via acting as interactive mediators regulating communication in the cells [11].

**Figure 1.**
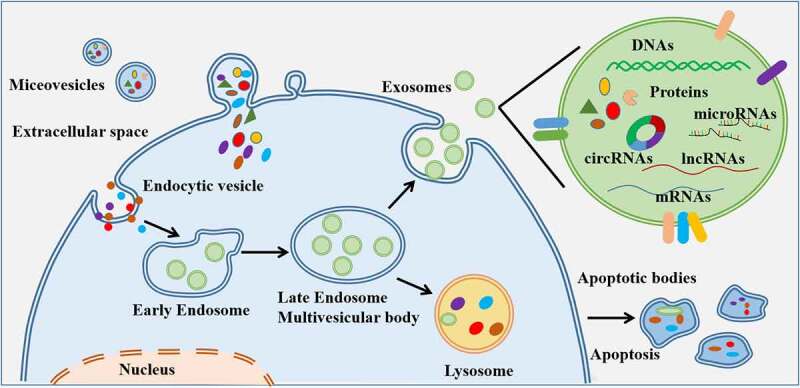
Secretion of extracellular vesicles (EVs) and their contents. EVs are originally derived from the endosomal and lysosomal pathway. EVs contain a range of proteins, RNAs, mRnas, DNA molecular cargoes, and surface protein markers. They can be released by any type of cells and can be transferred from the original cell to the recipient cells, into the extracellular microenvironment or to a distant site.

EVs are spherical bilayer-membrane vesicles which were secreted by almost all types of cell and released into the extracellular space [12]. According to the vesicle size (expressed in terms of the diameter), EVs can be classified as small extracellular vesicles (sEVs), also known as exosomes (30–100 nm), microvesicles (MVs, 100–1000 nm), and apoptotic bodies (1000–5000 nm) [13,14]. (Figure 1) Traditionally, exosomes are formed from invaginations in the endosomal compartments, which are known as multivesicular bodies and are secreted from the plasma membrane [14,15]. MVs are produced by direct budding from the plasma membrane, and apoptotic bodies are produced by the ‘blebbing’ of the plasma membrane in cells undergoing programmed death [16,17]. The bioactive molecules present in EVs include multiple proteins, DNA, mRNAs, non-coding RNAs, and metabolites. The presence of these bioactive molecules makes EVs critical contributors in the regulation of communications among tumor cells, tumor microenvironments, and distant organs and tissues [18–20]. In particular, sEVs were reported to play an important role in regulating tumor progression [21].

Increasing evidence indicates that the most important function of EVs in osteosarcoma progression is dependent on their role as intercellular transport systems. Moreover, the unique contents of EVs, such as non-coding RNAs, facilitate the use of exosomes as molecular markers for disease diagnosis [22]. Importantly, EV-based drug delivery systems have also been shown to be safe and efficient in cancer treatment [23,24]. In this review, we discuss existing knowledge about the important functions of tumor- or microenvironment-derived EVs on tumorigenesis, development, metastasis, drug resistance and communications with the osteosarcoma microenvironments. We also discuss the potential utility of exosomes as biomarkers in osteosarcoma diagnosis and novel strategies for osteosarcoma therapy.

## 2. Roles of tumor-derived EVs in osteosarcoma

**Figure 2.**
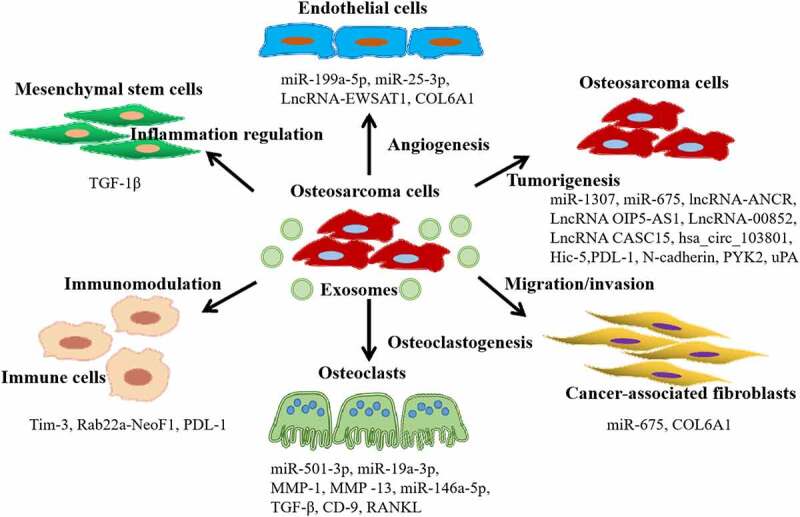
Functions of tumor-derived extracellular vesicles (EVs) in osteosarcoma progression and microenvironment. Firstly, tumor-derived EVs promote osteosarcoma tumorigenesis, and induce drug resistance through delivering multiple proteins, miRnas, and lncRnas to the recipient cancer cells. Secondly, tumor-derived EVs regulate the functions of endothelial cells and cancer-associated fibroblasts to promote angiogenesis and metastasis. Lastly, tumor-derived EVs also have significantly roles in osteoclastogenesis, inflammation regulation, and immunomodulation.

In recent times, increasing evidence has indicated that EVs play significant roles in the progression, proliferation, metastasis, anti-apoptotic effects, immune evasion, and chemotherapy resistance of osteosarcoma. These functions primarily rely on the influence of tumor-derived EVs on the tumor microenvironment and the proteins or non-coding RNAs transferred by tumor-derived EVs to recipient cells to regulate tumor metastasis, proliferation, and drug resistance. We summarized the tumor-derived EVs and the effects exerted on other cells in Table 1 and Figure 2.

**Table 1.** Overview of exosomal cargoes and functions of tumor-derived EVs.

Evs sources	Recipient cells	EVs cargos	Functions	Reference
Tumor cells	Tumor cells	hsa_circ_103801	Reduced the sensitivity of osteosarcoma cells to CDDP	[25]
Tumor cells	Tumor cells	LincTRNA-00852	Promoted proliferation, migration, and invasion	[26]
Tumor cells	Tumor cells	miR-1307	Promoted the proliferation, migration, and invasion	[27]
Tumor cells	Tumor cells	Hic-5	Inhibited apoptosis and increased proliferation	[28]
Tumor cells	Tumor cells	LncRNA OIP5-AS1	Promoted the angiogenesis	[29]
Tumor cells	Tumor cells	PDL-1, N-cadherin	Promoted pulmonary metastasis	[30]
Tumor cells	Tumor cells	lncRNA-ANCR	Induced drug resistance	[31]
Tumor cells	Tumor cells	miR-675	Promoted cell migration and invasion	[32]
Tumor cells	MSCs	-	Promoted bone microenvironment remolding	[33]
Tumor cells	MSCs	Transforming growth factor-β(TGF-β)	Promoted IL-6 release	[34]
Tumor cells	Tumor cells, Macrophages	PYK2, Rab22a-NeoF1	M2 type differentiation of macrophages and tumor lung metastasis	[35]
Tumor cells	Macrophages	-	Promoted the M2 phenotype and created an immunosuppressive, tumor-promoting microenvironment	[36]
Tumor cells	Macrophages	Tim-3	Induced M2 macrophage polarization and promoted the metastasis of osteosarcoma cells	[37]
Tumor cells	T cells	PD-L1	Induced immunosuppression	[38]
Tumor cells	BMDMs	miR-501-3p	Promotes osteoclastogenesis, aggravates bone loss	[39]
Tumor cells	Fibroblasts	COL6A1	Promote osteosarcoma cell invasion and migration	[40]
Tumor cells	HUVECs	miR-199a-5p	Inhibited tumorigenesis and angiogenesis	[41]
Tumor cells	Vascular endothelial cells	LncRNA-EWSAT1	Induced increase in angiogenic factor secretion and angiogenesis	[42]
Tumor cells	Endothelial cells	miRNAs	Potentiated tube formation, increased angiogenic markers expression	[43]
Tumor cells	Vascular endothelial cells	miR-25-3p	Promoted capillary formation	[44]

### 2.1. EVs in osteosarcoma tumorigenesis

An increasing number of studies have shown that EVs mediate the intercellular communications of tumor cells with other cells and play a significant role in tumorigenesis. For example, the osteosarcoma cell-derived exosomal miR-1307 promotes tumorigenesis via targeting AGAP1 [27]. What’s more, Zhang and colleagues reported that OS cell-derived exosomes convert normal fibroblasts to cancer-associated fibroblasts (CAFs) by secreting pro-inflammatory cytokines, including interleukin-6 (IL-6) and interleukin-8 (IL-8), and activated CAFs promote OS metastasis by regulating the TGF-β/COL6A1 signaling pathway [40]. Furthermore, Mu et al. reported that osteosarcoma-derived EVs activated NF-κB and Notch signaling in receipt muscle cells and induce the sarcoma-associated cachexia [45]. However, the study also showed that osteosarcoma cell-derived exosomal miR-199a-5p inhibited tumorigenesis by decreasing the expression of vascular endothelial growth factor A (VEGFA) [41].

### 2.2. EVs in osteosarcoma metastasis

The metastasis is an essential event in osteosarcoma progression. Gong et al. reported the high expression of exosomal miR-675 in metastatic OS cell lines compared with that in non-metastatic cells, and showed that the exosomal miR-675 decreased the calneuron 1(CALN1) expression and promoted the invasion and migration of recipient cells [32]. Bone marrow mesenchymal stem cell (BMSC)-derived exosomes express lncRNA-PVT1 at high levels, which can be transported to osteosarcoma cells; the transported PVT1 promotes tumor metastasis by sponging miR-183-5p, promoting the expression of ERG, and inhibiting the ubiquitination of ERG in osteosarcoma cells [46]. Moreover, tumor-derived exosomes induce M2 macrophage polarization, which in turn promotes the migration, invasion, epithelial-mesenchymal transition, and lung metastasis of osteosarcoma cells via the secretion of cytokines, including (interleukin-10, TGF-β, and VEGF [37]. Furthermore, Zhong and colleagues reveled that the exosomal Rab22a-NeoF1 fusion protein promotes the formation of the lung pre-metastatic niche by recruiting bone marrow-derived macrophages [35]. Additionally, osteosarcoma was shown to stimulate pulmonary metastasis by releasing exosomes that carry programmed death-ligand 1 (PD-L1) and N-cadherin [30]. Endo-Munoz et al. reported that metastatic osteosarcoma-derived EVs contain the urokinase plasminogen activator (uPA) and the uPA receptor (uPAR) at elevated levels, and that these EVs promote the conversion of non-metastatic osteosarcoma cells to a metastatic phenotype [47]. In addition, myeloid cell infiltration was shown to play a significant role in tumor metastasis, Mazumdar et al. demonstrated that osteosarcoma-derived EVs can recapitulate myeloid cell infiltration in the lungs but are unable to promote osteosarcoma metastasis [48].

### 2.3. EVs in osteosarcoma drug resistance

Per common knowledge, various factors contribute to the poor prognosis of osteosarcoma, the most important one being the resistance to conventional chemotherapeutic agents. The development of multi-drug resistance (MDR) forms a major obstacle in the improvement of the chemotherapeutic effects in osteosarcoma treatment [49]. The methotrexate-doxorubicin-cisplatin (MAP) regimen is a well-known chemotherapeutic strategy for osteosarcoma; however, cancer cells will gradually develop resistance to this regimen [50]. Recent findings show that cisplatin-resistant osteosarcoma cell (MG63/CDDP)-derived exosomes could be efficiently taken up by recipient MG63 and U2OS cells. Subsequently, circRNA_103801 was shown to expressed at high levels in MG63/CDDP-derived exosomes and could be transferred into recipient cells to reduce their sensitivity to CDDP, inhibit apoptosis, increase the expression of multidrug resistance-associated protein 1 and P-glycoprotein, and induce the establishment of the chemoresistance phenotypes [25]. Similar to these results, exosomes that contain high levels of MDR-1 and P-glycoprotein, derived from doxorubicin-resistant osteosarcoma cells, could be taken up by secondary cells and induce a doxorubicin-resistant phenotype in them [51]. Moreover, Liu et al. reported that CDDP-resistant osteosarcoma-derived EVs contain high levels of CCCTC-binding factor, which is transferred to osteosarcoma cells, enhances IGF2-AS transcription, and increases CDDP resistance in OS cells by regulating the IGF2-AS/miR-579-3p/MSH6 axis and activating an autophagy-dependent pathway [52]. What’s more, Hu et al. found that treating the treatment of doxycycline-sensitive KHOS/U2OS cells with exosomes isolated from doxycycline-resistant KHOS-DR/U2OS-DR enhanced tumor growth and progression, decreased overall survival, and induced resistance to treatment with doxycycline both in vitro and in vivo. Mechanistically, doxycycline-resistant-cell-derived exosomes transfer lncRNA-ANCR to induce drug resistance in osteosarcoma [31]. Furthermore, it has been reported that luteolin promote the effective packaging of miR-384 into the secreted exosomes of MG63 cells; these exosomes enhance cellular chemosensitivity to doxorubicin and cisplatin both in OS cells and xenograft models through inhibiting the PTN/β-catenin/MDR1 signaling axis [53]. In addition, Yati and colleagues reveled that doxorubicin treatment stimulated the release of EVs in osteosarcoma, and the recipient cells acquired chemotherapeutic resistance by the activation of IL-1/PD-L1 signaling [54].

## 3. Roles of tumor-derived EVs in the osteosarcoma microenvironment

In this part of the review, we will discuss the effects of EVs on the osteosarcoma microenvironment. The osteosarcoma microenvironment contains complex components, such as the extracellular matrix, osteoblasts, osteoclasts, endothelial cells, blood vessels, tumor-associated fibroblasts, and MSCs. As a carrier, EVs mediate the cellular communication in these cells. Jerez et al. performed the proteomic analysis of exosomes from human osteosarcoma cell lines and observed the secretion of more than 3000 proteins related to tumor progression that contributed to the communication between osteosarcoma cells and their microenvironment [55]. And Jerez et al. also showed that osteosarcoma cell lines may selectively package miRNAs as molecular cargo in EVs to modulate the tumor microenvironment [56].

### 3.1. Effects of tumor-derived EVs on MSCs

Recently, conditioned medium of osteosarcoma cells was reported to induce the carcinoma-associated fibroblasts (CAF)-like transformation of BMSCs. This result indicated the intercellular communication between osteosarcoma and BMSCs [57]. Mannerström et al. reported that osteosarcoma-derived EVs could modulate the fate of MSCs by modulating the epigenetic status and influence the expression of genes related to bone microenvironment remodeling [33]. Moreover, Baglio et al. reported that EVs secreted by highly malignant osteosarcoma cells selectively incorporate a membrane-associated form of TGF-β, which induced MSCs to produce the proinflammatory cytokine IL-6 [34]. Furthermore, Lagerweij et al. found that the systemic injection of human tumor EV-educated MSCs in mice bearing osteosarcoma xenografts strongly promoted cancer growth and metastasis formation by activating the IL-6/STAT3 signaling pathway [58].

### 3.2. Effects of tumor-derived EVs on osteoclast differentiation

Emerging evidence indicates that osteosarcoma cell-derived exosomes can be transferred to bone marrow-derived monocytes to promote osteoclast differentiation. Also, mechanistically osteosarcoma-derived exosomal miR-501-3p was shown to promote osteoclast differentiation and aggravate bone loss in vitro and in vivo via the PTEN/PI3K/Akt signaling pathway [39]. Moreover, Raimondi et al. showed that osteosarcoma cell-derived exosomes promoted osteoclast differentiation and bone resorption activity [43]. Luo and colleagues reported that osteosarcoma cell-derived EVs promoted osteoclast formation and enhanced bone resorption through transferring miR-19a-3p and regulating the PTEN/PI3K/AKT signaling pathway [59]. Furthermore, Garimella et al. demonstrated that extracellular membrane vesicles derived from osteosarcoma cells contain matrix metalloproteinases-1 and −13 (MMP-1, −13), TGF-β, CD-9, and receptor activator of nuclear factor-kappa B ligand in abundance, and promote osteoclastogenic and bone destruction [60]. In addition, Araki et al. reported that osteosarcoma-derived EVs decreased the number of mature osteoclasts in vivo and in vitro by transporting miR-146a-5p and suppressing the NF-κB signaling pathway [61]. Furthermore, Ucci et al. reported that EVs derived from osteosarcoma cells carried bone-related mRNAs and could be taken by osteoblasts and osteoclasts. They reduced osteoblast viability and activity, and increased TRAcP-positive area but did not alter osteoclastogenesis [62].

### 3.3. Effects of tumor-derived EVs on vascular endothelial cells and angiogenesis

Reportedly, MNNG/HOS cell-derived EVs contain transcripts of angiogenesis-related genes, such as angiopoietin 2, fibroblast growth factor 2 (FGF2), and VEGF genes, in abundance. These EVs induce tube formation from human umbilical vein endothelial cells (HUVECs) in vitro and angiogenesis in vivo, likely through a VEGF/ANGPT2/FGF2-mediated mechanism [62]. Exosomes-carrying EWSAT1 derived from osteosarcoma cells induced angiogenesis by increasing the sensitivity/reactivity of vascular endothelial cells and significantly promoted the proliferation, migration, colony formation, and survival of osteosarcoma [42]. Moreover, the osteosarcoma-derived exosome that enriched the lncRNA OIP5-AS1 regulates osteosarcoma tumor angiogenesis through miR-153 and ATG5 [29]. Additionally, exosomes derived from osteosarcoma cells showed remarkably higher levels of miR-199a-5p than HUVECs, and miR-199a-5p could significantly inhibited HUVEC proliferation, migration, and neovascularization by suppressing VEGFA expression [41]. Furthermore, osteosarcoma cell-derived exosomes potentiated tube formation from endothelial cells and increased the expression of angiogenic markers expression by inducing the specific packaging of miRNAs [43]. In addition, Yoshida et al. reported that tumor-derived exosomes promoted capillary formation and the invasion of vascular endothelial cells by regulating the miR-25-3p/WNT signaling pathway [44].

### 3.4. Effects of tumor-derived EVs on fibroblasts

In the tumor microenvironment, CAFs play an important role in cancer progression. Zhang and colleagues reported that OS cell-derived exosomes convert normal fibroblasts to CAFs by secreting pro-inflammatory cytokines, including IL-6 and IL-8 [40]. Moreover, exosomes derived from the metastatic but not non-metastatic osteosarcoma cells increase the migration and invasion of nonmalignant fibroblast cells via miR-675/CALN1 [32]. Furthermore, osteosarcoma-derived EVs induced a tumor-like phenotype in fibroblast cells, e.g. they enhanced the proliferation and survival of the cells under starvation, migration, adhesion, and 3D sphere formation [63]. In addition, Mazumdar et al. showed that osteosarcoma-derived EVs induce lung fibroblast reprogramming [64].

### 3.5. Effects of tumor-derived EVs on immune cells

Exosomes also play an important role in regulating the oncogenic molecular reprogramming of tumor-associated macrophages (TAMs). Recently, Wang et al. reported that osteosarcoma cell-derived exosomal ELFN1-AS1 mediated the M2 polarization of macrophages via the sponging of miR-138-5p and miR-1291 to promote the tumorigenesis in osteosarcoma [65]. Cheng et al. reported that osteosarcoma cells could induce the M2 type differentiation of macrophages largely through Tim-3 mediated by exosomes [37]. Moreover, Wolf-Dennen et al. reported that exosomes from metastatic osteosarcoma cells promoted transformation of the M2 phenotype in TAMs, thereby inducing the formation of an immunosuppressive, tumor-promoting microenvironment via the production of TGFB2 [36]. It has been reported that proline-rich tyrosine kinase 2 and Rab22a-NeoF1 fusion protein were sorted into osteosarcoma exosomes. The former induced the activation of Stat3 signaling in its recipient macrophages to promote the M2 phenotype, whereas the latter facilitated the formation of the pulmonary pre-metastatic niche by recruiting bone marrow-derived macrophages [35]. Furthermore, PD-L1-loaded exosomes extracted from osteosarcoma cells were reported to aggravate osteosarcoma progression by suppressing T cell activities [38].

## 4. Roles of tumor Microenvironment-derived EVs in osteosarcoma

**Figure 3.**
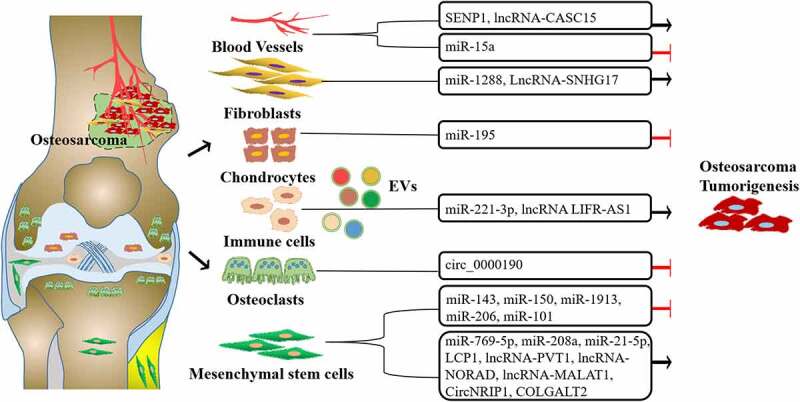
The regulatory network of microenvironment-derived extracellular vesicles (EVs) in osteosarcoma. EVs secreted in the plasma, immune cells, osteoclasts, chondrocytes, cancer-associated fibroblasts, and mesenchymal stem cells from different sources promoted or inhibited osteosarcoma tumorigenesis through different cargoes.

Exosomes derived from tumor cells mediate significant functions in intracellular communications. Similarly, tumor microenvironment-derived exosomes also play a critical role in osteosarcoma progression. Such as, Huang et al. showed that exosomes derived from BMSCs promoted osteosarcoma development by activating oncogenic autophagy [66]. Moreover, BMSC-derived exosomes inhibited osteosarcoma progression by transferring miR-206 and inhibiting transformer 2 protein homolog beta (TRA2B) [67]. In addition, Lu et al. reported that chondrocyte-derived exosomal miR-195 suppressed osteosarcoma cell proliferation and promoted cell apoptosis by targeting kinesin family member 4A in vitro and in vivo [68]. We summarized the tumor microenvironment-derived exosomes and their corresponding functions in Table 2 and Figure 3.

**Table 2.** Overview of exosomal cargoes and roles of microenvironment-derived EVs.

Fibroblasts	Tumor cells	miR-1288	Increased migration and invasion	[69]
Fibroblasts	Tumor cells	LncRNA-SNHG17	Promoted proliferation and metastasis	[70]
MSCs	Tumor cells	miR-143	Inhibited tumor cells migration	[71]
MSCs	Tumor cells	miR-150	Suppressed proliferation and migration	[72]
MSCs	Tumor cells	-	Activated PI3K/AKT and HIF-1α signaling pathway	[73]
BMSCs	Tumor cells	miR-1913	Inhibited tumor progression	[74]
BMSCs	Tumor cells	miR-206	Inhibited tumor progression	[67]
BMSCs	Tumor cells	miR-769-5p	Promoted proliferation and metastasis	[75]
BMSCs	Tumor cells	miR-208a	Promoted proliferation, invasion and migration	[76]
BMSCs	Tumor cells	miR-21-5p	Promoted proliferation and invasion	[77]
BMSCs	Tumor cells	-	Promoted oncogenic autophagy	[78]
BMSCs	Tumor cells	(lymphocyte cytosolic protein 1) LCP1	Promoted tumorigenesis and metastasis	[79]
BMSCs	Tumor cells	lncRNA-PVT1	Promoted cell growth and metastasis	[46]
BMSCs	Tumor cells	-	Activated the hedgehog signaling	[80]
BMSCs	Tumor cells	lncRNA-NORAD	Promoted migration, invasion, and angiogenesis	[81]
BMSCs	Tumor cells	lncRNA-MALAT1	Promoted proliferation, invasion and migration	[82]
BMSCs	Tumor cells	CircNRIP1	Promoted tumor progression	[83]
ADMSCs	Tumor cells	COLGALT2	Fostered cell invasion, migration and proliferation	[84]
ADMSCs	Tumor cells	miR-101	Suppressed tumor progression	[85]
Macrophages	Tumor cells	lncRNA LIFR-AS1	Promoted proliferation, invasion, suppressed apoptosis	[86]
Macrophages	Tumor cells	miR-221-3p	Aggravated cell growth and metastasis	[87]
Plasma	Tumor cells	SENP1	-	[88]
Plasma	Tumor cells	lncRNA-CASC15	Promoted tumor progression	[89]
Plasma	Tumor cells	miR-15a	Suppressed proliferation and invasion	[78]
Osteoblast	Tumor cells	circ_0000190	Suppressed tumorigenesis	[90]
Osteoblast	Tumor cells	-	Regulated mineralization process	[91,92]
HUVECs	Tumor cells	-	Promoted the stemness of osteosarcoma cells	[93]
Chondrocyte	Tumor cells	miR-195	Inhibited proliferation and anti-apoptotic	[68]

### 4.1. Role of CAFs-derived EVs in osteosarcoma

First, CAFs can affect cancer progression of cancer by releasing exosomes. Wang et al. reported that CAF-derived exosomes promoted cell migration and invasion in osteosarcoma cells by delivering miR-1228 to directly inhibit SCAI expression [69]. Moreover, CAFs promoted the proliferation and metastasis of osteosarcoma cells by transferring lncRNA-SNHG17 to sponge miR-2861 and then promoted MMP2 expression in osteosarcoma cells [70]. Apart from fibroblasts, the tumor microenvironment also contains osteoblasts and osteoclasts. Li et al. showed that osteoblast-derived extracellular nanovesicles transmitted the circular RNA has_circ_0000190 to osteosarcoma cells, which subsequently suppressed the migration, proliferation, invasion, and biological malignant behavior of the tumor cells [90]. Agnieszka et al. revealed that human osteoblast hFOB 1.19-derived EVs affect the mineralization [91,92].

### 4.2. Effects of TAMs-derived EVs in osteosarcoma

TAMs are r important regulators in the tumor microenvironment with a critical role in tumorigenesis regulation. For example, the conditioned medium of TAMs induced epithelial-mesenchymal transition and promoted the lung metastasis of osteosarcoma [94]. Recently, miR-221-3p derived from M2-polarized TAM exosomes was reported to promote proliferation and metastasis and aggravate the malignancy of osteosarcoma by regulating the SOCS3/JAK2/STAT3 axis [87]. What’s more, Zhang showed that macrophage-derived exosomes could facilitate osteosarcoma cell progression both in vitro and in vivo and revealed the mechanism by which exosomal lncRNA-LIFR-AS1 promoted osteosarcoma cell proliferation and invasion, and inhibited cell apoptosis via the miR-29a/NFIA axis [86]. In addition, lncRNA-CASC15 was shown to be overexpressed in osteosarcoma plasma exosomes and promote osteosarcoma progression through the regulation of the miR-338-3p/RAB14 axis [89].

### 4.3. Effects of MSCs-derived EVs in osteosarcoma

MSCs also form an important component of the tumor microenvironment. For example, adipose-derived MSCs were shown to secrete exosomes to foster growth and metastasis of osteosarcoma cells by increasing collagen beta (1-O) galactosyl transferase 2 expression [84]. Under stress, MSCs stimulate osteosarcoma migration and apoptosis resistance via EV-mediated communication [95]. Additionally, MSC-derived EVs promoted the proliferation of osteosarcoma cells by regulating PI3K/AKT and HIF-1α expression under hypoxia [73]. Moreover, BMSC-derived exosomes activated the hedgehog signaling pathway in recipient osteosarcoma cells and increased cancer cell migration and promoted tumor growth [80]. Huang et al. showed that exosomes derived from BMSCs promote osteosarcoma cell proliferation, migration, invasion, and development by activating oncogenic autophagy [66]. BMSC-derived exosomes, which express the lncRNA PVT1 in abundance, promoted osteosarcoma growth and metastasis by regulating the miR-183-5p/ERG axis [46]. The BMSC-EV-derived lncRNA-NORAD promoted migration, invasion, and angiogenesis in osteosarcoma cells by regulating the expression of CREB-binding protein via the delivery of miR-877-3p [81]. BMSC-derived EVs promoted the proliferation, invasion, and migration of osteosarcoma cells via the lncRNA MALAT1/miR-143/Neurensin-2 (NRSN2)/Wnt/β-catenin axis [82]. And it has been reported that circNRIP1 encapsulated by BMSC-derived EVs aggravates osteosarcoma by modulating the miR-532-3p/AKT3/PI3K/AKT axis [83]. The exosomal transfer of miR-769-5p from BMSCs to osteosarcoma cells promoted the proliferation and metastasis by targeting dual-specificity phosphatase 16 [75]. Furthermore, BMSC-derived exosomes contained miR-21-5p or miR-208a in abundance, which promoted osteosarcoma cell proliferation, migration, and invasion by targeting PI3KR1 or programmed cell death protein-4, respectively [76,77]. In addition, BMSC-derived exosomes carrying proteins, such as LCP1, stimulated osteosarcoma metastasis via the LCP1-induced activation of the JAK2/STAT3 signaling pathway [79].

Conversely, MSC-derived exosomes exhibited tumor suppressor function in osteosarcoma. Such as, Zhang and colleagues reported that BMSC-derived exosomes inhibited the proliferation, migration, and invasion of osteosarcoma cells and induced their apoptosis both in vitro and in vivo by delivering miR-206 and inhibiting TRA2B expression [67]. Similarly, Zhou and Xu showed that BMSC-derived exosomes inhibited osteosarcoma progression by transferring miR-1913 or miR-150 to osteosarcoma cells to suppress NRSN2 or IGF2BP1 expression, respectively [72,74]. Moreover, Keremu et al. reported that EVs from BMSCs transported microRNA-206 into osteosarcoma cells and targeted NRSN2 to block the ERK1/2-Bcl-xL signaling pathway, thus inhibiting OS progression [96]. In addition, EVs derived from adipose tissue-derived mesenchymal stromal cells delivered miR-101 to osteosarcoma cells and suppressed the lung metastasis of tumor cells by inhibiting BCL6 expression [85].

## 5. Role of EVs in the diagnosis and therapeutics of osteosarcoma

### 5.1. Role of EVs in the diagnosis of osteosarcoma

In recent years, liquid biopsy has been used to assess the molecular heterogeneity of tumors declared a new and efficient strategy for cancer diagnosis. EVs, owing to their stability and ease of isolation, is a novel candidate for liquid biopsies. EVs contain lots of proteins, non-coding RNAs and DNA, and these components may serve as effective biomarkers for osteosarcoma. Recently, Cambier et al. reported that EVs contained lots of repetitive-element DNA, including HSATI, HSATII, LINE1-P1, and Charlie 3, and their overexpression in EVs was associated with a receiver operating characteristic (ROC) area under the curve (AUC) ≥0.90. These results demonstrated that EV-associated repetitive element DNA could be used as candidate osteosarcoma biomarkers [97]. What’s more, Bao et al. reported that the RNA sequencing of EVs revealed drastic transcriptomic alterations between metastasis and primary osteosarcoma in a liquid biopsy approach [98]. Xu et al. reported that mRNAs, including those of Annexin2, Smad2, MTAP, CIP4, Cdc5L, and P27, were differentially expressed in the exosomes of patients with osteosarcoma with different chemotherapeutic responses, indicating that exosomal RNAs are reliable biomarkers for classifying osteosarcoma with different chemotherapy sensitivities [99].

Apart from DNA and mRNAs, dysregulated non-coding RNAs in EVs may serve as effective biomarkers for osteosarcoma. Such as, using high-throughput sequencing, Ye et al. identified that 57 miRNAs, 20 of which were upregulated and 37 downregulated, were differentially expressed in patients with osteosarcoma and healthy controls. These microRNAs exhibit potential as biomarkers for the diagnosis of patients with osteosarcoma [100]. Moreover, Cuscino and colleagues showed the presence of eight novel microRNAs, which were selectively packed into the exosomes of osteosarcoma cell lines. Five of the eight novel microRNAs were present more abundantly in the circulating exosomes of patients with osteosarcoma than in controls, indicating the role of these eight novel microRNAs in osteosarcoma diagnosis [101]. Agarwal et al. used next-generation sequencing and Q-RT-PCR to show that the upregulated canine exosomal Cfa-miR-9 could act as a bispecific osteosarcoma markers [102]. What’s more, Yoshida and colleagues reported that miR-25-3p, which was expressed at a high level in tumor-derived exosomes, exhibited clinicopathological relevance and could potentially serve as a noninvasive biomarker in osteosarcoma [44,103]. Furthermore, Huo and Dou revealed that circ_0056285 was highly expressed in serum exosomes of patients with osteosarcoma, and the AUC of the ROC curve was 0.778, indicating that the level of exosomal circ_0056285 had high diagnostic value in osteosarcoma [104]. In addition, lncRNA CASC15 was shown to be upregulated in osteosarcoma plasma exosomes compared with that in control exosomes, and its expression has been confirmed to predict poor prognosis in human cancers [89,105].

Studies also indicated that EVs derived proteins can also function as potential biomarkers for predicting prognosis of osteosarcoma. Han’s group used SERS and MALDI-TOF MS to elucidate the distinctive biochemical differences in the spectra of plasma-derived exosomes of 15 patients with osteosarcoma and 15 healthy volunteers. This approach can be used for the rapid identification of osteosarcoma [106]. And their group also identified seven plasma exosomal proteins as potential biomarkers for the lung metastasis of osteosarcoma [107]. Moreover, Weinman et al. used mass spectrometry to show that exosome proteomic signatures correlate with chemotherapy resistance and carboplatin treatment outcomes in a spontaneous model of canine osteosarcoma [108]. And Jacqueline et al found that ten exosomal proteins showed significant differential expression in serum of osteosarcoma patients and healthy dogs, with an accuracy of 85% for discriminate. Additionally, serum samples isolated at different disease stages could be distinguished with an accuracy of 77% based on the exosomal proteomic composition [109]. Wang reported that the plasma exosome SENP1 levels were related to tumor size, tumor location, necrosis rate, pulmonary metastasis, and surgical stage. Both disease-free survival (DFS) and overall survival (OS) were worse in patients with higher SENP1 levels than in patients with lower SENP1 levels [88]. In addition, patients with the pulmonary metastasis of osteosarcoma were shown to have a relatively higher level of exosomal PD-L1 than patients without metastasis, and the AUC value of the ROC curve was 0.823 for exosomal PD-L1, which indicated the pulmonary metastasis progression for OS patients [30]. Besides, Luu et al. recently reported that the proteomic analysis of extracellular vesicle preparations from canine tissue explants revealed 29 proteins that are significantly upregulated in OS-derived vesicles compared to that in normal bone-derived vesicles. According to their results they pointed out that PSMD14/Rpn11could be a molecular target of the drug capzimin in osteosarcoma [110].

### 5.2. Role of EVs in the therapeutics of osteosarcoma

Exosomes are an efficient delivery system for non-coding RNAs and could be used for osteosarcoma therapeutics. Such as, BMSC-derived exosomes could be engineered and serve as efficient nanocarriers. Several studies have shown the efficient antitumor function of BMSC-derived exosomes by carrying non-coding RNAs, including miR-206 [67], lncRNA-PVT1 [46], and miR-101 [85]. For example, synthetic miR-143 transfected into MSCs could be released as exosomes form and be transferred to osteosarcoma cells, and then inhibited the migration of the osteosarcoma cells [71]. Moreover, Xue and colleagues reported that miR-371b-5p-engineered exosomes can be effectively internalized by tumor cells, and their overexpression was shown to promote cell apoptosis, inhibit cell proliferation and migration, and suppress osteosarcoma formation in vivo [111]. Huang et al. prepared the engineered exosomes cRGD-Exo-MEG3 and found that it could deliver lncRNA-MEG3 more efficiently to osteosarcoma cells both in vitro and in vivo, and have potentially therapeutic effects for osteosarcoma [112]. What’s more, the novel nanodrug, Exo-Dox, is an MSC-derived exosome loaded with doxorubicin; Exo-Dox has been proved have enhanced cellular uptake efficiency and anti-tumor effect in osteosarcoma [6]. Furthermore, Notaro et al. found that WIN, a synthetic agonist of cannabinoid receptors, suppressed osteosarcoma cell migration by increasing the release of EVs and dramatically upregulating miR-29b1 [113]. Wang et al. reported that bevacizumab, an anti-vascular endothelial growth factor, suppressed exosomal lncRNA-MIAT derived from serum-derived EVs transferred to osteosarcoma cells. And then arrested osteosarcoma cell proliferation and angiogenesis by inducing miR-613-mediated GPR158 inhibition [114]. In addition, Qin et al. reported that luteolin, a flavonoid compound, inhibited the multidrug resistance of osteosarcoma cells through increasing the packaging of miR-384 into exosomes and then suppressing the PTN/β-catenin/MDR1 signaling axis [53].

## 6. Conclusion

In this article, we discussed the role of EVs in osteosarcoma and their potential in tumor diagnosis and treatment. However, several challenges in the use of EVs remain unaddressed. First, more accurate and standardized purification methods are needed for isolating EVs from the body fluids of patients with osteosarcoma. Second, further studies are needed to figure out which cargoes that serve as the main functional components. Most importantly, more effective and safer engineered EVs should be developed for drug delivery. In conclusion, a thorough understanding of EVs will help develop better EVs-based diagnosis and treatment strategies for osteosarcoma treatment.

## Data Availability

Data sharing not applicable to this article as no datasets were generated or analyzed during the current study
